# Using the United Kingdom standards for public involvement to evaluate the impact of public involvement in a multinational clinical study

**DOI:** 10.1186/s40900-021-00264-3

**Published:** 2021-04-30

**Authors:** Kathy Seddon, Jim Elliott, Miriam Johnson, Clare White, Max Watson, Annmarie Nelson, Simon Noble

**Affiliations:** 1 Marie Curie Palliative Care Research Voices, Cardiff University Research Partner, Cardiff, Wales UK; 2 Health Research Authority, London, UK; 3 Wolfson Palliative Care Research Centre, Hull York Medical School, University of Hull, Hull, UK; 4 Northern Ireland Hospice, Belfast, Northern Ireland UK; 5 Hospice UK, London, UK; 6 Marie Curie Palliative Care Research Centre, Cardiff University, Heath Park Campus 8th Floor Neuadd Meirionydd, Cardiff, Wales CF14 4YS UK

**Keywords:** Patient public involvement, UK standards, Venous thromboembolism, Lay representative

## Abstract

**Background:**

The publication of the United Kingdom (UK) Standards for Public Involvement (PI) (UK Standards) in research drew a clear line in the sand regarding the importance of utilising the unique experience, skills and expertise that lay people may offer to the development, conduct and dissemination of clinical research. The UK Standards provide a benchmark which researchers should aim to achieve, yet its implementation continues to be a step wise iterative process of change management. A recent evaluation by a regional research group has suggested that our understanding of PI is enhanced through reflection on the UK Standards. We report on the utility of PI in the design, conduct and dissemination of the HIDDen study, a national, multicentre clinical study based across three UK centres.

**Methods:**

A retrospective review of PI within the HIDDen study was conducted using field notes taken by the lead author from interactions throughout their involvement as a lay representative on the study. Key members of the HIDDen study were interviewed and data analysed to explore adherence to the UK Standards.

**Results:**

There was universal support for PI across the study management group with genuine inclusivity of lay members of the committee. All six of the UK Standards were met to varying degrees. The greatest opportunities lay in ‘working together’ and ‘support and learning’. There were challenges meeting ‘governance’ with evidence of participation in decision making but less evidence of opportunities in management, regulation, leadership.

**Conclusion:**

This study concurs with previous research supporting the utility of the Standards in the conduct and evaluation of PI in clinical research. To our knowledge this is the first multi-national study to be evaluated against the UK Standards.

**Supplementary Information:**

The online version contains supplementary material available at 10.1186/s40900-021-00264-3.

## Plain English summary

The past decade has seen a genuine increase in patient and public involvement (PI) in clinical research, far beyond a symbolic presence on a trial management committee or inclusion on a grant application. The United Kingdom (UK) Standards for Public Involvement provide a useful structure to support PI throughout a study as well as defining a benchmark that can be used to improve the involvement of patients and the public in studies.

The importance of reflecting on and reporting on PI in specific studies has been recognised since it contributes to a stepwise change process which will eventually lead to PI becoming normal practice for clinical research. A recent review identified a myriad of frameworks by which PI may be evaluated, risking an inconsistent approach to PI evaluation and consequently slowing down its progression.

The Hospice Inpatient Deep vein thrombosis Detection study (HIDDen) was a national multicentre study to explore the prevalence and associated variables of blood clots in patients with advanced cancer when they were admitted to the specialist palliative care unit.

In this paper we will be considering the HIDDen research in terms of the UK Standards for Public Involvement.

## Background

The role of patient and public involvement (PI) is increasingly recognised as a vital component in conducting high quality, rigorous medical research [1]. The United Kingdom’s (UK) National Institute of Health Research (NIHR) defines PI in research as:

*“Research being carried out* ***‘****with or ‘by’ members of the public rather than* ***‘****to****’, ‘****about****’*** *or* ***‘****for****’*** *them. This may include working with research funders to prioritise research, offering advice as members of a project steering group or co-applicant, commenting on and developing research materials and undertaking interviews with research participants, co-authoring papers and dissemination."*

Recent literature has highlighted the need for more work to evaluate and thereby normalise the PI contribution to research [2]. A “step change” approach has been suggested, whereby PI will eventually become “business as usual” [3]. Several enablers to successful PI have been highlighted including flexible research methodology, shared values and putting goals and rationales first [4, 5].

The UK Standards for Public Involvement (referred to hereafter as the UK Standards) were established in order to set a quality benchmark, which all research activity should aspire to meet [6]. The process by which the UK Standards were developed is described in detail by Crowe and colleagues [7]. Initial development comprised of an evaluation of existing standards, with the majority of content derived from those previously published by INVOLVE and Health Care Research Wales [8]. A synthesis of these features formed the basis of the first draft which comprised six standards with accompanying indicators. This document was then subjected to a rigorous 4 month consultation period involving the public, academics, charities and involvement practitioners. Through this process, the standards were modified, with particular changes involving the decision to remove the indicators for each standard. They were updated to the UK Standards for Public Involvement and published in November 2019 following a further pilot phase [9]. They highlight six key values of involvement, namely: respect, support, transparency, responsiveness, fairness of opportunity, and accountability [10]. Such standards and values will optimise the gains that can be achieved from PI, particularly the co-production of knowledge, if well planned support systems and patient centred technology are used [3, 10]. As with any aspect of research reporting, it is important to ensure consistency in detailing PI involvement in research. However, there are at least 65 reporting frameworks currently published and consistency is unlikely to be achieved until a consensus is reached on which framework should be used [11]. Furthermore, it has been suggested that the breadth of “off-the-shelf” published frameworks have limited transferability and stakeholders might consider co-designing their own frameworks based on a menu of evidence-based resources.

Mathie and colleagues recently reported on the utility of the UK Standards within a regional research network [12]. Although the UK Standards enhanced the research process, they noted that some studies fail to routinely integrate and report PI into the main results paper, or as a separate paper. Several constraints to PI were identified including the time lapse to involvement before funding is confirmed and payment issues across organisations.

In contribution to the “step change” suggested by Galvin and colleagues, this paper shall review PI in an NIHR funded, multinational multi-centred clinical study, against the UK Standards [8, 13].

### Context: the HIDDen study

The Hospice Inpatient Deep vein thrombosis Detection study (HIDDen), recently published in the Lancet Haematology, was a multicentre, prospective, longitudinal, observational study to explore the prevalence, symptom burden and natural history of venous thromboembolism (VTE) in people with advanced cancer [13]. It was funded by the NIHR Research for Patient Benefit programme (PB-PG-0614-34,007). VTE is when a blood clot is formed in the deep veins, usually the leg, causing what is known as a deep vein thrombosis (DVT). Part of this clot may break off and travel to the lungs forming a pulmonary embolus. Cancer patients are at a particularly high risk of VTE since the cancer itself and anti-cancer treatments increase the stickiness of the blood. One in five cancer patients will develop VTE and the risk increases with advancing disease [14]. Guidelines recommend that hospitalised medical patients (including those with cancer) receive primary thromboprophylaxis (blood clot preventers) in the form of an injectable medicine low molecular weight heparin [15]. However, it was not known whether these guidelines should apply to patients with advanced cancer, when admitted to hospices and specialist palliative care units (SPCU) since the thromboprophylaxis clinical trials excluded patients with poor prognosis or performance status [16]. Furthermore, the rationale for routine thromboprophylaxis is based on improving overall survival and not the prevention of distressing symptoms. The utility of such outcomes and such research has been questioned by palliative care healthcare professionals. The aim of the HIDDen study, therefore, was to explore the true prevalence of DVT in cancer patients admitted to hospices and SPCUs and to evaluate the impact VTE had on their quality of life. The study involved recruiting cancer patients admitted to one of five SPCU/hospices in England, Wales and Northern Ireland, to undergo a bedside ultrasound to evaluate the presence or absence of DVT. These data were correlated with clinical symptoms, blood results and clinical outcomes to better understand the true impact of VTE in patients with advanced cancer.

The original (draft) National Standards were first launched whilst HIDDen was ongoing although PI had been facilitated through the patient charity Thrombosis UK.

### Links to comparable studies

The HIDDen study was managed through Hull York Medical School (the University of Hull), with colleagues in Cardiff University working within a well-developed PI framework, taking the lead for PI. Following the original launch of the National Standards, the group devised a PI review tool, basing its questions on the on indicators suggested within the first draft of the Standards. This tool, developed under the auspices of the Wales Cancer Research Centre (WCRC) can be viewed in Additional file 1. It should be noted that these were developed at a time that the UK Standards were in their first draft, when the indicators were still included. For this reason, this paper also has also cited the indicators to illustrate the derivation of some of the questions. The review tool essentially uses questions drawn from each of the standards. For example, for INCLUSIVE OPPORTUNITIES we asked. “Were public involvement opportunities offered that are accessible and that reach people and groups according to research needs?”

### Theoretical rationale and influences -concepts and theory development

We report on the integration and development of PI within the HIDDen study through its use of the UK Standards for Public Involvement as benchmarks. The standards are designed to be a description of what good PI in research looks like. They are designed to encourage the kind of self-reflection and learning central to this review. The six UK Standards are defined as:

- INCLUSIVE OPPORTUNITIES: Offer public involvement opportunities that are accessible and that reach people and groups according to research needs.
- WORKING TOGETHER**:** Work together in a way that values all contributions, and that builds and sustains mutually respectful and productive relationships.
- SUPPORT & LEARNING**:** Offer and promote support and learning opportunities that build confidence and skills for public involvement in research.
- COMMUNICATIONS**:** Use plain language for well-timed and relevant communications, as part of involvement plans and activities
- IMPACT**:** Seek improvement by identifying and sharing the difference that public involvement makes to research
- GOVERNANCE**:** Involve the public in research management, regulation, leadership and decision making

We have used these standards as a framework for reporting a retrospective review of PI in the HIDDen study.

### Aim of study

A retrospective review of PI in the HIDDen study to identify good PI practice in the light of the UK Standards for Public Involvement in research.

## Methods

### People involved

The people involved in the study are listed, together with an outline of their role.

- The Principal Investigator was *Professor Simon Noble* who has great experience and expertise in this field.
- Academic Expertise was provided by *Professor Annmarie Nelson* who is the Scientific Director, Marie Curie Palliative Care Research Centre Cardiff; and Academic Lead for Public Involvement and Engagement in the School of Medicine.
- The Research Nurse *Becky Cloudsdale* was based in the Marie Curie Hospice in Penarth and interacted directly with patients there*.*
- The Nurse Manager of the In-Patient Unit at the Marie Curie Hospice Penarth, where the research was carried out, was *Ceri Davies*. Ceri is a nurse of over 15 years experience and has completed her master’s research at Cardiff University with Professor Anne Marie Nelson.
- Public Contributor 1(PC1) *Annya Stephens-Boal*
- Public Contributor 2 (PC2) *Dr Kathy Seddon. (Lead author)*
- Public Contributor 3 (PC3) *Content expert and paper review Dr Jim Elliott*

Annya has had lived experience, having lost her mother to a pulmonary embolism and suffered from recurrent VTEs herself. She is Executive Officer at Thrombosis UK and interacts regularly with people who experience thrombosis. This is in line with the Health Research Authority’s (HRA) principle of ‘involving the right people’ bringing valuable experiences and skills that are relevant and important for this research. Kathy is a long serving member of Marie Curie’s research voices and a Research Partner at the Wales Cancer Research Centre. Jim has experience in the field of PI (HRA) and has made a significant contribution to the paper.

The co-Chief Investigators *(Prof Miriam Johnson and Dr Clare White)* and the rest of the research team recognised the importance of PI. Public contributors reported being treated as respected members of the research team,

### Design, data capture

As part of her role within the trail management group, PC2 undertook a recruitment site field visit to evaluate how PI had impacted on the research process as well as identifying further opportunities for PI to contribute. As part of this process she met informally with the Principal Investigator, the Research Nurse, who recruited hospice patients to the study and the Nurse Manager at the hospice. The body of work was classed as part of a service/ process evaluation and did not require ethical approval.

She undertook informal interviews to explore the impact of PI throughout the study, and to provide insights about the importance of each of the UK Standards in guiding the research. Interviews were anonymised with only field notes kept as a record of discussions. The interviews were guided by a semi-structured prompt list, based around the UK Standards, which had been developed and piloted through the WCRC. For example, under the standards ‘COMMUNICATIONS’ we asked about the use plain language for well-timed and relevant communications, as part of involvement plans and activities. The WCRC PI tool can be viewed in Additional file 1.

Contemporaneous field notes were taken from each interview and collated for a report by PC2 and a project officer supporting PI research. Field notes contained no quotes or identifying characteristics. The report aimed to establish how PI was used at different stages of the HIDDen project and to draw up a timeline of PI. The write up of the interviews were shared with interviewees for comment, rigour and validation and forms the basis for this paper.

## Study results

The importance of each of the UK Standards in the HIDDen project are considered below in general terms and then more specifically in the timeline table that follows. The UK Standards are seen as ‘interacting cogs’ so the numbers do not imply a hierarchy.

### One: INCLUSIVE OPPORTUNITIES: offer public involvement opportunities that are accessible and that reach people and groups according to research needs

It was confirmed that PI input took place at the very earliest stages of the HIDDen project. This included help in formulating the research question, in the research prioritisation process and in considering the acceptability of the study intervention (PC1). Public contributors (PC1 and PC2) were members of the study research team. Additional public contributors were sought via a publicly advertised expression of interest outlining the role but received no interest. Opportunities to be involved were signalled through local networks such as the Involving People Network in Wales and a full range of media was used both for information and then participation.

Choice and flexibility in the PI opportunities were offered. Involvement could be remote or face to face and contributions were included in meeting minutes at document review meetings and governance discussions. It was felt that PI representatives were encouraged to develop points of interest including suggestions for post project feedback. The input to the documentation by PC1 proved invaluable in recruiting informed and diverse participants.

Looking at barriers to people getting involved, making the recruitment process as effective as possible was felt to be important. Public contributors need to feel safe and free to speak honestly. An understanding was reached that ‘baggage’ from past experience isn’t detrimental. It was however vital that it was channelled, so that all contributions were effective, and none dominated. This was felt to have been achieved, since team discussions were free flowing with PC1 and PC2 contributing widely and productively. A key to achieving this lay with team members having a shared understanding of each other’s roles goal and the purpose of PI within the research project. It also benefitted from strong leadership by the chair of the trail management group to ensure inclusion of PI members at all times.

### Two: WORKING TOGETHER work together in a way that values all contributions, and that builds and sustains mutually respectful and productive relationships

The team felt that the purposes of PI were jointly defined though they were not a set of prescriptive written rules. The public contributors were partners and team members. They were secure in their representative role.

Team dialogues involved an empowering iterative interaction, with respect on both sides. They allowed improvements in patient information and consent documentation to ensure where possible recruitment of diverse and informed participants. PI was not just symbolic; public contributors took an active part in discussions and questioning at meetings. They had feedback on their roles and contributions, which evolved over time. All meetings were fully documented, and joint decisions recorded. Some of the key contributions were in adding a lay voice to patient information and consent documentation to ensure where possible diverse and informed participants. The decision to allow proxy consent, for patients lacking capacity due to the temporary reversible complications of cancer, came about through this dialogue after a suggestion by PC1. This was an important development since causes of loss of capacity, including infection, opioid toxicity, hypercalcaemia are not only reversible but will also temporarily increase the risk of VTE. These patients are the ones most likely to benefit from thromboprophylaxis and it is essential for them to be represented in the study.

### Three: SUPPORT & LEARNING offer and promote support and learning opportunities that build confidence and skills for public involvement in research

Resources needed for PI were identified at an early stage of planning. They were monitored and adjusted as necessary. For instance, researchers were allocated core time to work with the public contributors, which was flexible enough to meet changing requirements. The provision of facilities for public contributors with additional needs was carefully considered and offered as needed. Public contributors were offered Good Clinical Practice (GCP) training where relevant. Strictly speaking, GCP training was not necessary for HIDDen, since it was not a clinical trial of an investigational medicinal product. However PC1 took the opportunity for further training in order to have a broader appreciation of the responsibilities of researchers. The online GCP material was reviewed for information and useful links for further reading noted. Public contributors were given additional training to ensure their informed input to matters of ethical compliance through online training materials which were followed up with one to one discussions with the Principal Investigator. Provision was made for public contribution at workshops and at conferences relevant to the HIDDen study. This included presentation at national cancer conference in a symposium on PI in research as well as participation in a round table meeting about future directions for VTE research.

### Four: COMMUNICATIONS use plain language for well-timed and relevant communications, as part of involvement plans and activities

A plan for flexible communication was in place using a range of media. Various media opportunities indicated in the UK Standards were used, including newsletters, Twitter, website news, conference statements and annual reports. Feedback from public contributors and the rest of the team was gathered to be shared and learned from. The review tool was created collaboratively and the interviews at which it was used resulted in clearly communicated information about the process and value of the PI in the research.

### Five: IMPACT seek improvement by identifying and sharing the difference that public involvement makes to research

The impact of PI was a key area considered and one in which public contributors were involved in reporting, auditing and recording all examples of impact. A review was written to inform future PI in new research. One key point that emerged was that early PI is essential in this iterative, interactive research process. The agreed purpose for PI and its intended outcomes were detailed in the original grant application as a basis for impact review. This then drove the creation of a series of reports for different audiences such as funders. Valuable lessons for best practice were identified in these reports including engaging with PI at the ethics stage and using PI to disseminate results through academic, stakeholder and guideline development meetings. This was an important clinical study with practice changing implications and careful recording of key activities and benefits was therefore put in place.

### Six: GOVERNANCE involve the public in research management, regulation, leadership and decision making

Public contributors were invited to join the study management group and were included in the project organisational organograms. Reports to funders detailed PI activities and these activities were reviewed regularly. Funding for PI was assured as it was embedded in the grant application with standard travel and an honorarium included if desired. However, based on the authors observations and field interviews, PI activities were limited with respect to governance issues.

In summary, in terms of PI, the study involved a complex interaction between public contributors, including patients and carers, with the study researchers and hospice staff. The excellent management of this interaction has produced the impacts and benefits highlighted in this report.

The table below summarises the details of PI in the HIDDen research study as a timeline. It highlights the outcomes and benefits that accrued and suggests where the UK Standards might have been a useful benchmark. For convenience in the table, numbers will be used as a shorthand to refer to the standards though the UK Standards model shows them as ‘interdependent cogs’.

**Table:** 

Summary of PI in the HIDDen research project			
Date	Event	Outcomes and benefits of PI in this research	PI Standards indications
*Summer 2014 to 2016 Pre implementation planning*			
June 2014	Input by PC1 at an early team meeting to discuss acceptability of study design and clinical intervention	Highly praised input to study deign and acceptability of bedside compression ultrasonography giving the lay perspective.	Standards one, two and six. The team enabled involvement in formulating the research. Working together in management group
March 2016	Planning input at group phone conference by PC1	On important suggestion was to allow proxy consent when necessary that was immediately adopted. This had a profound beneficial effect on data collection	Standard two. Key decision taken after public contributor advice
September 2016	Input to documentation through email by PC1	Modification of wording in the lay summary and research documentation. This greatly improved accessibility for the public contributors	Standards one, two and four. PI at earliest stage. Joint wording in lay summary. Plain language ensured
September 2016	Input to recruitment through email discussion with PC 1	PC1’s approach to recruitment ‘widened’ the participation though ensuring that it remained within capacity. PC1 aimed to recruit ‘informed’ people by giving them sufficient facts in an understandable form in the documentation. This was very successful in ensuring inclusivity and a diverse group of contributors	Standards one and four. Inclusive opportunities for PI. Working together toward clear material for communication
*Spring/summer 2017 Implementation Phase*			
30th June 2017	PC 2, who was asked to review PI activity, took part in an initial meeting with the local research team to review the whole timeline of research in terms of the UK Standards	The HIDDen research study was outlined to the second public contributor (PC2) who subsequently co-produced the review tool set of detailed questions* about all PI at each stage of the research. The tool questions probed the research – through the UK Standards. PC2 produced an outline report which was discussed and finalised at a subsequent meeting**	Standards four and five. The difference PI has made has been fed back, captured and communicated
13th Dec 2017	PC 2 later met the Principal Investigator and the research nurse at local recruitment site (Hospice)**	Further details were explored relevant to PI in the HIDDen study. Some of the review questions needed input from the research nurse and nurse manager of the hospice. The finalised tool proved flexible and useful for wider research purposes in other projects	Standard two learning (research nurse) Standard six potential PI in conferences planned
19th Jan 2018	PC 2 again met Principal Investigator, the research nurse and nurse manager at the hospice	Further questions were answered about all of the PI in the HIDDen research. This allowed a full final report to be written.	All standards: Agreed purpose for PI and intended outcomes detailed in original grant application - as a basis for impact review and for a series of reports for different audiences such as funders
*Spring 2018 / 9 Review phase*			
Spring 2018	Both public contributors (based at different sites) agreed dissemination input	Analysis of the study from the PI perspective was completed and a final report completed with further possible PI in the dissemination phase indicated. The report forms the basis for this paper	Standard five. Further involvement in dissemination and impact
Spring 2019	HIDDen research continued to be reviewed in terms of The UK Standards	Further Interviews with Principal Investigator to assess the HIDDen research in the light of the newly released **UK Standards** for Public Involvement in research were undertaken	Standard five impact. Full review of HIDDen research in relation to UK Standards
Summer 2019	Data relating to the revised UK Standards analysed for reporting	Team collaboration was excellent	Standard five reviewing PI impact collaboratively

#### Highlighting some key benefits in the HIDDen research resulting from PI

- Proxy consent suggestion had potential effect on data collection.
- Document accessibility to a wider audience resulted in improved data collection
- Informed participants recruited to HIDDen thanks to PI input to creation of clear documentation.
- Review tool based on National Standards co-produced and piloted.
- Finalised review tool based on UK Standards used to provide the basis for this paper.
- Dissemination of findings about HIDDen and the UK Standards allows consideration and comparison of PI in other research.
- Collaborative and respectful ways of working established that became the norm for valued collaboration in future research.

## Discussion and conclusions

We have reported on the successful, multi-factorial aspects of PI in a national multi-centre clinical research study, focusing on the impact the public contributions made to the design, conduct, and reporting of the study. We have demonstrated that the PI work in the research study can be considered in terms of the UK Standards, which were introduced during the course of the study. It is hoped that this paper adds to the increasing body of literature highlighting examples of good practice demonstrating the value of PI in clinical research.

In achieving the aims of this paper, we have used the UK Standards for public involvement in research, through a purpose designed review tool to ensure adequate coverage of key detail of the research process.

There are several issues to be considered:

### Utility of UK standards useful in evaluating the use of PI in the HIDDen research

The UK Standards provide thorough and useful guidelines of how research should be structured around PI. In terms of evaluating a research study that had started before the UK Standards were finalised, retrospective recall may however lack detail about all the valuable co-construction that occurred.

There is some potential overlap in the UK Standards since PI activity may ‘fit’ into two or more of the standards. This makes it difficult to use them in a review of a completed complex piece of research. It is however possible to recognise possible areas where the standards were not met. For instance, whilst it is straightforward to note the involvement of PC1 to the research question, project management groups and organograms: there is no evidence to suggest any involvement in the study design. Thus, standards one (opportunities) and six (governance) suggest this might be a possible area for improvement.

It was not possible to evaluate all standards within a finite period of time. Even when the research has been completed some actions were ongoing. For instance, standard five (impact) is a rich and evolving tapestry of PI actions that are still being implemented.

It was straightforward to document examples of working together (standard two) and the mutual respect and team ethos came across clearly. The standards will undoubtedly enhance this in future pieces of research.

There were many instances of support and learning (standard three). Unpicking the range offered could perhaps be helped if the standards highlighted that there is a spectrum to be considered here; from simple training (documented) to capture of PI input for joint authorship of conference papers and posters. Perhaps one of the best documented examples of the UK Standards in this research is in the abundant evidence of good communication (standard four).

Contribution to the Research Ethics Committee (REC) paperwork and attendance in REC meetings is a growing area of PI involvement in a number of research projects. Whilst help with ethics paperwork was documented, attendance at the REC meeting was not.

The UK Standards thus provide a basis from which further action might evolve. Given that the HIDDen study was ongoing during the release of the UK Standards the PI was commendable. Consideration of the UK Standards in research is increasing as researchers see potential benefits that can accrue though using them as a guide in future projects.

### How does PI in the HIDDen project fits into the wider picture of PI in research?

A recent systematic review by Greenhalgh and colleagues identified 65 frameworks for supporting patient and public involvement in research [11]. They proposed five categories of framework or types of PI focus:

- Power-focused; designed to surface, explore and overcome researcher-lay power imbalances. Their theory underpins many of the other frameworks.
- Priority-setting focus: designed to involve patients and lay people in setting research priorities e.g. James Lind Alliance
- Study-focused: principles and methods for involving patients and lay people in conducting research, building a culture of involvement at all stages of the research cycle. Thereby improving the quality and efficiency of research and maximizing its societal impact e.g. UK Standards for Public Involvement in research and the earlier NIHR Research Handbook 2014 which illustrates PI in terms of the research cycle. This is the basis of this paper
- Report-focused: designed to guide writing up and critical appraisal of research reports; a checklist for critically appraising a published study for the quality and comprehensiveness of patient and lay involvement.
- Partnership-focused: designed to assure transparency and public accountability in researcher-lay collaborations. e.g. the INVOLVE values and principles. This sets out the principles of 1. Respect 2. Support 3. Transparency 4. Responsiveness 5. Fairness of opportunity 6. Accountability. They are to a large extent included in the UK Standards where INVOLVE had a key role and have thus already been considered

They suggested the breadth of “off-the-shelf” published frameworks have limited transferability and that stakeholders consider co-designing their own frameworks based on a menu of evidence-based resources [11]. They concluded that “a single one size-fits-all framework may be less useful than a range of resources that can be adapted and combined in a locally generated co-design activity”. In this paper, three of the listed frameworks (study, report and partnership focussed) have been incorporated to create a fitting basis for exploration. As suggested, these frameworks might be used selectively by stakeholders to co-design their own frameworks. For this review the study-focused framework allowed consideration of:

- The research context and nature of the proposed study
- The planning for involvement including the resources needed alongside the UK Standards for PI
- How the research went beyond tokenism – ensuring that PI is more than “ticking a box”
- Inclusivity and human aspects such as building relationships, clarifying roles, communicating clearly, establishing trust and sharing information
- The development and nurturing of an ongoing relationship with lay partners

We have reported the impact of PI in a major multi-site research study using the frameworks of the UK Standards for PI. The HIDDen study successfully used aspects of all UK Standards as a benchmark. This is a useful case study to further inform clinical researchers wanting to work effectively with public contributors. The UK Standards highlight partnerships-focused values and principles and a consideration of respect, support, transparency, responsiveness and accountability when working with public contributors.

### Limitations to the project and lessons learned

This was the first time the UK Standards have been used to evaluate PI in a multi-national clinical study and from the start, it was always expected to be an iterative processes. Limitations to the project were inevitable and should be viewed within the wider context of what the project has achieved.

Firstly, the decision to evaluate HIDDen PI against the UK standards was a bold one since they were still in development and undergoing modifications. Such an example was the use of indicators, which were removed from the final draft of the standards but present at the time we undertook our evaluation. Has been evaluated, Secondly, the review was retrospective and some detail was inevitably lost since it relied on recall and some meeting documentation was unavailable as mentioned. Future studies within our group shall look to evaluate PI prospectively. Finally, it should be acknowledged that the evaluation reflects many interpretations and judgements on the part of the first author which risks bias. Every effort was made to limit this through he review of data by members of the trial management group in order to bring other perspectives to the interpretation of the data.

In considering lessons learned, the most significant is the importance of ensuring the UK Standards are used at the outset of the research project. Ideally this should be at stage of developing the research question. This would ensure full value for PI involvement. In the context of HIDDen study specifically, the evaluation concluded that PI did not meet the standards with respect to GOVERNANCE. In future studies we would aim to improve PI opportunities in research management, regulation and leadership.

## Conclusions

Our findings therefore concur with Mathie and colleagues in their Review of Regional working in the East of England: using the UK National Standards for Public Involvement. They suggest that “*as more reflective papers are published and the National Standards are more widely used in the UK, many lessons can be learnt and shared on how to improve our Patient and Public Involvement within research studies. Evaluations or reflections such as these can further enhance our understanding of PI with implications for regional, national and international comparisons.”* [11] Since that paper was published, the UK Standards were finalised [6]. Nonetheless the key messages remain the same and are endorsed by our work. Hopefully, this will be another vital step in highlighting the benefits of well-planned PI in research.

## Supplementary Information

**Additional file 1.**

## Data Availability

On request.

## Abbreviations

DVT: Deep Vein Thrombosis
HRA: Health Research Authority’s
HIDDen: Hospice Inpatient Deep vein thrombosis Detection study
GCP: Good Clinical Practice
NIHR: National Institute of Health Research
PC: Public Contributor
PI: Public Involvement
REC: Research Ethics Committee
SPCU: Specialist Palliative Care Unit
UK: United Kingdom
VTE: Venous Thromboembolism
WCRC: Wales Cancer Research Centre
