# Supplementary material for: Using the United Kingdom standards for public involvement to evaluate the impact of public involvement in a multinational clinical study
Source: Res Involv Engagem. 2021 Apr 30;7:22. doi: 10.1186/s40900-021-00264-3 (PMC8088001; doi:10.1186/s40900-021-00264-3)
Supplement: Supplementary file 1 — Additional file 1. [file 40900_2021_264_MOESM1_ESM.pdf]

**Standard 1: Inclusive Opportunities**<br>We offer public opportunities that are accessible and that reach people and groups according to research needs		
Indicator 1.1	We involve people affected by and interested in research topic/issue at the earliest stage	
<u>Prompt</u>	Are public contributors involved in:	❑ Trial/study design<br>❑ Research prioritisation processes<br>❑ Formulating the research question
Indicator 1.2	We identify and address barriers to taking up public involvement in research	
<u>Prompt</u>	Are there particular challenges in involving public contributors effectively e.g. payment, nature of research, ability to travel, use of IT, accessing diverse communities or other.	
	What solutions have been found to these challenges?	
Indicator 1.3	We make information about opportunities for public involvement available, using different methods so that we reach relevant and interested people	
<u>Prompt</u>	From where are public contributors drawn?	❑ Local PPI networks e.g. Involving People (Wales)<br>❑ Existing or active groups<br>❑ Recruited from specific communities. Please give examples.
	How might people find out about involvement opportunities?	❑ Websites<br>❑ Direct email<br>❑ Twitter<br>❑ Radio<br>❑ Word of mouth<br>❑ Other
Indicator 1.4	We have a fair and transparent recruitment processes for involving the public in research	
<u>Prompt</u>	The recruitment process includes:	❑ Role descriptions<br>❑ Person specifications<br>❑ Adverts<br>❑ Interviews<br>❑ Recruiter feedback post applications
Indicator 1.5	We offer choice and flexibility in opportunities for public involvement in research	

© 2018 (copy with permission) Professor Annmarie Nelson, Dr Kathy Seddon, Kate Cleary

Prompt	Is there a choice of types of opportunities for public involvement, e.g.: meetings, reviewing documents, governance?	Please list opportunities:
	Is there flexibility in undertaking public involvement opportunities e.g.: working remotely, attending meetings via teleconference	Please list:
	Are public contributors free to identify and develop projects of interest?	❑ Yes<br>Can you give an example:

**Standard 2: Working Together**<br>We work together in a way that values all contributions, and that builds and sustains mutually respectful and productive relationships		
Indicator 2.1	We jointly define and record the purpose of our public involvement activity	
Prompt	Is there a role description to fit the involvement activity?	❑ Yes<br>❑ No
	Are objectives set per involvement activity?	❑ Yes<br>❑ No
Indicator 2.2	We develop public involvement plans and activities together	
Prompt	Do public contributors take part in reviewing, developing and coordinating involvement activities?	❑ Yes<br>❑ No
Indicator 2.3	We ensure there is a shared understanding of roles, responsibilities and expectations, which may evolve over time	
Prompt	Are public contributors offered an individual annual review?	❑ Yes<br>❑ No
	Are public contributors offered the chance to contribute to a report identifying progress within the PPI work?	❑ Yes<br>❑ No
	Is there a document that sets the mutual expectations of the role and responsibilities?	❑ Yes<br>❑ No
	Are researchers asked about public involvement in progress meetings or annual review?	❑ Yes<br>❑ No
Indicator 2.4	We recognise individual ideas and contributions and uphold decisions together	
Prompt	Are suggestions implemented as a result of public involvement inputs?<br>Are actions recorded?	❑ Yes<br>❑ No<br>❑ Yes<br>❑ No

© 2018 (copy with permission) Professor Annmarie Nelson, Dr Kathy Seddon, Kate Cleary

Standard 3: Support and Learning<br>We offer and promote support and learning which builds confidence and skills for public involvement in research		
Indicator 3.1	We designate and monitor resources to ensure and support effective public involvement	
Prompt	Is there a system for financial support for public contributors?	❑ Yes<br>❑ No
	Is there dedicated public involvement support/resource for researchers? E.g. admin support/academic lead for public involvement.	❑ Yes<br>❑ No
	Are researchers given core time to undertake public involvement in their role?	❑ Yes<br>❑ No<br>❑
Indicator 3.2	We offer a range of support to address identified needs	
Prompt	Is access provided to people with additional needs such as mobility issues, carer support, or learning difficulties?	❑ Yes<br>❑ No
	Please give examples of support given.	
Indicator 3.3	We have a clearly identified point of contact for information and support	
Prompt	Is there a named lead for public involvement within research teams/staff groups?	❑ Yes<br>❑ No
	Do public contributors have a named mentor?	❑ Yes<br>❑ No
Indicator 3.4	We develop, deliver and monitor learning opportunities in partnership, for all involved in research	
Prompt	Is information about accessing training for public contributors provided? E.g. from local public involvement networks	❑ Yes<br>❑ No
	Is ad hoc training, provided by researchers, available to public contributors?	❑ Yes<br>❑ No
	Is public involvement training available to researchers?	❑ Yes<br>❑ No
	Have standard operating procedures on areas such as recruitment, training, supporting and financing public involvement been developed?<br>Are researchers trained and updated on standard operating procedures?	❑ Yes<br>❑ No<br><br>❑ Yes<br>❑ No

© 2018 (copy with permission) Professor Annmarie Nelson, Dr Kathy Seddon, Kate Cleary

Indicator 3.5	We actively learn from others, we build on what we have learned and share our learning	
Prompt	Do researchers:	❑ Attend conferences/workshops?<br>If yes, how many in the last 12 months________<br>❑ Present at conferences/workshops?<br>If yes, how many in the last 12 months________<br>❑ Do they publish aspects of their work with public contributors?<br>❑ Engage with other groups working with public involvement.

**Standard 4: Communications**<br>We use plain language for timely, two way and targeted communications, as part of involvement plans and activities		
Indicator 4.1	We develop and deliver a communications plan for our involvement activities	
Prompt	Are different media platforms used to communicate about public involvement plans and activities?	❑ Annual report<br>❑ Newsletter<br>❑ Website presence<br>❑ Blog<br>❑ Twitter<br>❑ Word of mouth<br>❑ Other
Indicator 4.2	We are inclusive and flexible in our communication methods to meet the need of different people	
Prompt	Is information available in different formats such as easy to read format or translated upon request (Wales)?	❑ Yes<br>❑ No
Indicator 4.3	We gather, offer and act on feedback, which we then share	
Prompt	Is feedback invited from public contributors?	❑ Yes<br>❑ No
	Is feedback shared and acted upon?	❑ Yes<br>❑ No<br><br>If yes, how?

© 2018 (copy with permission) Professor Annmarie Nelson, Dr Kathy Seddon, Kate Cleary

Standard 5: Impact<br>To drive improvement, we capture and share the difference that public involvement makes to research		
Indicator 5.1	We involve the public in the assessment of public involvement in research	
Prompt	Are public contributors involved in reporting, auditing, impact recording, or review processes?	❑ Yes<br>❑ No
	Are the outcomes of these assessments acted on?	❑ Yes<br>❑ No<br><br>If yes, please provide an example:
Indicator 5.2	We record our agreed purpose for public involvement and its intended outcomes	
Prompt	Is there an agreed policy detailing purposes of public involvement and intended outcomes?	❑ Yes<br>❑ No
	Are there set objectives per involvement activity?	❑ Yes<br>❑ No
Indicator 5.3	We collect information that will help us assess the impact of public involvement in research	
Prompt	Are public involvement activities collated and reported against objectives?	❑ Yes<br>❑ No<br><br>If yes, how? e.g. online diaries
Indicator 5.4	We reflect, learn and report the extent to which we have met our intended purpose and predicted outcomes	
Prompt	Are public involvement specific outcomes reported?	❑ Yes<br>❑ No<br><br>If yes, how are outcomes reported:
	Is the impact of public involvement recorded?	❑ Yes<br>❑ No<br><br>If yes, how is impact recorded:
	Are best practice case studies collected and reported?	❑ Yes<br>❑ No
	Are valuable lessons from best practice examples acted upon?	❑ Yes<br>❑ No<br>If yes, how is impact recorded:

© 2018 (copy with permission) Professor Annmarie Nelson, Dr Kathy Seddon, Kate Cleary

**Standard 6: Governance**<br>We involve the public in our governance and leadership so that our decisions promote and protect public interest		
Indicator 6.1	Public voices are heard, valued and included in decision making	
Prompt	Are the public involved in setting research questions via:	❑ Priority setting exercises<br>❑ Steering groups<br>❑ Executive groups<br>❑ Research management groups<br>❑ Funding committees
Indicator 6.2	We have public involvement strategies and/or plans in place that we regularly monitor, review and report on	
Prompt	Are public involvement activities included in reports to funders?	❑ Yes<br>❑ No
	Is public involvement policy and objectives reviewed on an annual basis?	❑ Yes<br>❑ No
Indicator 6.3	Responsibility for public involvement is visible and accountable through our management structure	
Prompt	Are public contributors invited to join management groups?	❑ Yes<br>❑ No<br>If yes, at what level? e.g. executive/senior/project
	Are public contributors visible in organisational structures e.g. included on organograms?	❑ Yes<br>❑ No
Indicator 6.4	We allocate money and other resources for public involvement	
Prompt	Is there specific funding for public involvement?	❑ Yes<br>❑ No
	From where does public involvement funding originate?	
	Does the public involvement budget include costs for:	❑ Staff<br>❑ Volunteers<br>❑ Involvement activities<br>❑ Senior leadership
	Is there communications support available for public involvement?	❑ Yes<br>❑ No

© 2018 (copy with permission) Professor Annmarie Nelson, Dr Kathy Seddon, Kate Cleary
